# Therapeutic community drug treatment success in Peru: a follow-up outcome study

**DOI:** 10.1186/1747-597X-3-26

**Published:** 2008-12-03

**Authors:** Knowlton Johnson, Zhenfeng Pan, Linda Young, Jude Vanderhoff, Steve Shamblen, Thom Browne, Ken Linfield, Geetha Suresh

**Affiliations:** 1 Pacific Institute for Research and Evaluation, Inc., 1300 S. Fourth Street, Ste. 300, Louisville, KY 40208, USA; 2 Bureau for International Narcotics and Law Enforcement Affairs, U.S. Department of State, 2201 C Street NW, Washington, DC 20520, USA; 3 Spalding University, 845 S. Third Street, Louisville, KY 40203, USA; 4 Justice Administration, University of Louisville, Louisville, KY 40292, USA

## Abstract

**Background:**

The purpose of this study was to assess the impact of drug abuse treatment in Peru that used the therapeutic community (TC) model. Program directors and several staff members from all study treatment facilities received two to eight weeks of in-country training on how to implement the TC treatment model prior to the follow-up study.

**Methods:**

This outcome study involved 33 TC treatment facilities and 509 former clients in Lima and other cities in five providences across Peru. A retrospective pre-test (RPT) follow-up design was employed in which 30-day use of illegal drugs and alcohol to intoxication was measured at baseline retrospectively, at the same time of the six-month follow-up. In-person interview data were collected from directors of 73 percent of the eligible TC organizations in January and February 2003 and from former 58 percent of the eligible TC former clients between October 2003 and October 2004. Drug testing was conducted on a small sample of former clients to increase the accuracy of the self-reported drug use data.

**Results:**

Medium to large positive treatment effects were found when comparing 30-day illegal drug and alcohol use to intoxication before and six months after receiving treatment. As a supplemental analysis, we assumed the 42 percent of the former clients who were not interviewed at the six month assessment had returned to drugs. These results showed medium treatment effects as well. Hierarchical Generalized Linear Modeling (HGLM) results showed higher implementation fidelity, less stigma after leaving treatment, and older clients, singly or in combination are key predictors of treatment success.

**Conclusion:**

This study found that former clients of drug and alcohol treatment in facilities using the TC model reported substantial positive change in use of illegal drugs and alcohol to intoxication at a six-month follow-up. The unique contribution of this study is that the results also suggest attention should be placed on the importance of implementing the TC drug abuse treatment model with fidelity. Further, the results strongly suggest that TC drug abuse treatment programs should incorporate follow-up activities that attempt to neutralize community negative reactions (perceived stigma) independent of other factors.

## Background

### Therapeutic community drug treatment research in perspective

Much has been written about the underlying principles of the therapeutic community (TC) drug abuse treatment model (e.g., [1,2]). Woodhams[2] describes this model as one in which staff members interact with residents in an environment where "community as method" is the organizing principle. This community is assumed to be responsible for one another's treatment, having an equal role in the treatment process rather than staff bearing the primary responsibility for treatment. The staff role is to facilitate the implementation of TC principles by behavior modeling and to reinforce the community concepts and philosophy in the community's decision-making process. Staff and residents share the following concepts and beliefs:

#### ▪ View of the disorder

Addiction problems are centered within the individual; physiological symptoms exhibited are secondary.

#### ▪ View of the person

Treatment plans are individually tailored; however, addicts often share certain characteristics, such as low self-esteem, lack of impulse control, low tolerance for frustration, inability to cope with feelings, dishonesty, poor interpersonal skills, immaturity, and feelings of being a victim.

#### ▪ View of recovery

Residents must learn experientially through feedback from encounter groups and interaction with other residents in the community to recognize and change negative behavior.

#### ▪ View of right living

Residents are encouraged to adopt a philosophy that reinforces moral and ethical beliefs typically held by mainstream society rather then continuing to hold negative, self-serving views. Residents are encouraged to own their feelings and internalize pro-social feelings of doing what is right in a given situation.

In addition to clinical and administrative staff, TCs often employ staff members who are ex-users or graduates of a TC program[2]. The recovering staff members are considered "rational authorities" who use their skills and experiences to guide, teach, coach, and correct negative behavior of clients[3]. The role of the staff is to facilitate the development of clients' treatment plans by coaching, reinforcing corrective and positive behavior, clarifying issues, and lending assistance when needed. Staff interaction with drug addicts in a therapeutic community is more intense than in other treatment milieus; it is more frequent and can be more confrontational.

This article reviews the research on TC drug abuse treatment and training and presents results of a large TC drug treatment follow-up study in Peru. The study focuses on the treatment success of 33 treatment facilities in various locations of the country and predictors of treatment success. Multi-level analysis procedures were employed to take into consideration the bias due to the influence of a facility on all former clients from that facility. This sample of 33 facilities is a subsample of 72 TC facilities that were involved in a study of Daytop TC training impact on staff[4]. The purpose of the current study is to ascertain changes in former clients' use of alcohol and other drugs after treatment in TC facilities and to determine whether predictors of client changes in alcohol and other drug use after treatment could be identified. Evaluation of drug treatment effects in developing countries is scarce. This study is one of the first studies to document what happens to former clients after drug treatment in a developing country.

### Studies of TC drug abuse treatment effects on client behavior

Over the past 30 years an unprecedented number of applied addiction treatment outcome studies have been conducted. Major catalysts for this research have been the National Institute on Drug Abuse (NIDA) national research programs: Drug Abuse Reporting Program (DARP) in the 1970s[5,6]; Treatment Outcome Prospective Study (TOPS) a decade later[7]; in the 1990s, the Drug Abuse Treatment Outcome Studies (DATOS)[8,9]; and the National Treatment Outcome Research Study (NTORS)[10]. A consistent body of research has supported the effectiveness of drug treatment in general (e.g., [9]) and of TCs in particular[11,12].

Criticisms of drug treatment outcome studies have pointed to methodological shortcomings that investigators regularly debate. In a review of drug treatment outcome methodology reported in peer-reviewed journals between 1993 and 1997, Ellingstad and colleagues[13] found that less than one-fourth of the articles used a minimum six-month follow-up interval, which is an important consideration because the highest period of relapse has been found to be between three and six months following treatment[14].

Client outcome studies of the therapeutic community model have focused on specific target populations that the programs are intended to serve (e.g., prison or jail inmates, chronically homeless drug or alcohol abusers, youth/adults in the general population with chronic and debilitating drug/alcohol problems, dual diagnoses of mental illness and drug addiction) and have employed a variety of methodologies (pre, post tests, single case study, comparative studies, etc.). The outcomes that are typically measured are related to the most serious problems associated with these groups (e.g., re-arrest or reconviction rates, unstable living arrangements and employment, lack of reduction or abstinence from drugs or alcohol use).

The single case study method has often been used to examine outcomes of TC treatment clients in a particular program. For example, Wilson and Mandelbrote[15] examined reconviction rates of ex-residents of the Ley Community TC in Oxford, England, and found correlations with historical patterns of criminal behavior, prior drug use, and length of duration in the program. The authors concluded that TC treatment is effective in reducing criminal activity for residents who stay in the program longer than six months. Holland[16,17] found similar results for residents of the Gateway House in Illinois. In a five-year follow-up study of ex-residents of the Phoenix House program in New York, De Leon[18] also found improvements related to duration in the program. Dekel and colleagues[19] found that fifteen months after leaving the TC program, half of the Israeli heroin addict participants were clean. Those who had lived with a partner before entering the TC and those who had not engaged in theft prior to treatment were more likely to be drug free at follow-up. A 12-month follow-up study of 83 graduates from community-based TC programs found the majority of participants reported being free from alcohol- and illegal drug use as well as experiencing improvements in the areas of employment, living arrangements, family relationships and high-risk negative behaviors[20].

Using a pre- and post-test design, De Leon[21] found that both graduates and dropouts of the Phoenix House program improved significantly on measures of personality disorder and self-esteem at the two-year follow-up but client improvements were still below the "normative" or healthy range. Ravndal[22] collected pre and post data from program applicants, dropouts, and completers of a Phoenix House program in Oslo, Norway, and found that completers had fewer substance abuse problems and better social functioning outcomes at the five-year follow-up than those who never entered or dropped out of the program. Those reporting lower frequency of drug use before applying or entering the program had higher rates of success at the five-year follow-up. In a 12-month follow-up of 83 (70%) graduates of inner-city TC programs, researchers found the majority still abstaining from drug and alcohol use, and experiencing improvements in employment, living arrangements, family relations, criminal and other high-risk negative behavior[20]. In a comparative study of two TCs and two no-treatment groups in the Netherlands, Kooyman[23] found much better outcomes for the TC clients at six-month follow-up. De Leon and colleagues[24] found greater behavioral improvements at the 12-month follow-up among homeless, mentally ill chemical abusers in two modified TC programs than those assigned to the control group.

### Predictors of favorable treatment outcomes

Studies have consistently shown the length of time a drug user stays in a treatment program is one of the most important predictors of successful treatment outcomes[25,11,28]. The effectiveness of treatment programs is limited by the TC's ability to retain the clients for a period long enough to promote change. Typically, relatively few stay beyond three months[29]. Early treatment follow-up studies [30-32] found that successful client outcomes related to reduced crime and substance use and increased employment were related to time spent in treatment. Gossop and colleagues[33] reported that critical time in treatment (28 days for shorter in-patient programs and 90 days for longer-stay rehabilitation programs) was strongly correlated with improvement in overall drug use, and that those who stay in the programs past the critical times were more than five times as likely to have achieved abstinence from all target drugs at the one-year follow-up than those who left. In a comparison of standard and abbreviated treatment in a TC treatment, De Leon[25] and Nemes and colleagues[34] found that positive outcomes are associated with "graduation" or completion of the entire treatment regimen, regardless of the length of the program. Toumbourou and colleagues[35] concluded that attainment of level progress is a better predictor of treatment outcomes.

Chan and colleagues[36] found that treatment satisfaction is related to pre-treatment problem severity and duration of treatment. More recent studies have found that program dropouts are more likely to have had conflicts with the program's rules and view the program as punishment, while completers tend to view the program as treatment and have more positive evaluations of staff[37-39]. Further, Carlson and Gabriel[40] found that client satisfaction with access and effectiveness was associated with six-month follow-up service utilization, as well as one-year post-treatment abstinence from drugs. Kasarabada and colleagues[41] found that only two perceived characteristics of therapists, nurturance and openness, showed significant correlations with length of stay in treatment. However, positive perceptions of counselors had no significant effect on reducing drug use severity scores at a one-year follow-up.

The extent to which a program adapts the ideal TC model and its essential elements, and how staff actually implements the model, has been of interest to investigators (e.g., [42,43]). Some attention, although limited, has been given to defining program fidelity operationally. Prendergast and colleagues[44] found in a meta-analysis study that well-implemented TC drug abuse treatment and outpatient drug-free programs were correlated with more positive behavioral outcomes. A number of studies have found positive direct effects of the quality and quantity of program implementation on drug-related outcomes [45-48]. Hansen and colleagues[49] found program fidelity to be a moderator of substance abuse prevention program effectiveness.

Some TCs provide additional staff training and education to build both staff skills in group dynamics and understanding of TC philosophy and ethos[2]. Mistral and colleagues[50] found that applying the principles of the therapeutic community to a high-care psychiatric ward did improve staff communication and staff attitudes. Focus groups with TC staff revealed that even after training on TC theory, methods, and procedures, staff have a vague idea about their roles and responsibilities in a TC setting and felt that experiential learning is important in working with drug addicts[51].

In a study of the impact of staff training to implementing structural family therapy in an adolescent therapeutic community, Weidman[52] concluded that increased staff confidence and competence may result in fewer dropouts and increased attendance in family therapy. Johnson, Young, Suresh, and Berbaum[4] conducted a three-year intervention study using a social policy experiment design that employed a randomized design with repeated measures to test hypotheses about the effects of TC training conducted in Peru. The study tested for the direct and moderating effects of the training, examined implementation fidelity, and reexamined the underlying theory of the TC training model. The study found that the vast majority of the staff participants reported positive appraisals of the quality of trainers (e.g., trainers explained things), the quality of training content and methods (e.g., training handouts were helpful), the quality of training environment (e.g., training rooms and facilities were comfortable and convenient), and cultural sensitivity (e.g., cultural issues were handled with respect). The researchers concluded that the quality of training implementation was judged as more than adequate since *a priori *expectations, as set by the trainers, were exceeded for all appraisal criteria. Further, the study found that the training had medium effects on staff behavior outcomes such as implementation fidelity of TC tools and small effects on staff empowerment to use TC methods and tools and actual use of TC principles.

McMillin[53] reports many former drug addicts feel shame and guilt years after their last drink or drug use. He further states that stigma is a primary obstacle to solving our nation's alcohol and drug problems. A few studies have looked at the impact of stigmatization on drug use and treatment. Furst and colleagues[54] suggest that the shame and stigma associated with the label "crackhead" served as a deterrent to potential adolescent users. Falck and colleagues[55] found that stigma associated with cocaine use can serve as a barrier to treatment and use of clinical and non-clinical services. In a study of the relationship between methamphetamine use and depression, Semple and colleagues[56] found that perceived stigma had a significant positive direct effect on depressive symptoms above and beyond that accounted for by methamphetamine use. Although we could find no studies that examined the relationship between outcomes of TC clients and their perception of social "stigma," studies of patients in mental hospitals have suggested that labeling and social stigma are related to treatment outcome variables such as self-esteem, employment status and social networks[57,58]. Room[59] advocates for both quantitative and qualitative studies that examine potential preventive effects of stigmatization.

### Therapeutic communities in Peru and treatment outcomes

As in most developing countries of the world, drug addiction is a serious social problem in Peru[60]. Beginning in the mid-1970s, the United Nations Fund for Drug Abuse Control (UNFDAC) received contributions from the Government of Italy to establish Therapeutic Community (TC) treatment centers throughout Peru. Although a network of TCs were subsequently created in Peru and other South American countries by UNFDAC, treatment reports (outcome evaluations, etc.) in Latin America were scarce, mainly focusing on epidemiological studies[61]. Furthermore, there were no systematic treatment reports in the Peruvian literature, and the initial papers only addressed treatment guidelines[61]. Beginning in 1978, Navarro[62,63] described the treatment and follow-up of only two patients dependent on coca paste; both patients subsequently stopped using the drug. In 1980, Sanchez[64] published a study on the treatment of 50 drug addicts at the Nana TC center in Lima, of which 15 addicts eventually became abstinent. Several years later, Navarro and colleagues[65] conducted a study on 26 clinical patients at Nana TC who were followed-up from 8 months to 4 years after treatment. From 1982 through 1989, Navarro[61] conducted a larger study of 223 male patients of the Nana TC, who were mainly consumers of coca paste. Over 47 percent were abstinent at follow-up.

Although UNFDAC had assisted in the creation of a network of TCs in Peru, the Peruvian government determined in 1997 that many of these programs were poorly trained, providing inadequate services, and needed to be licensed by the government. In 1997, this urgent need for TC drug abuse treatment training prompted the Peruvian government to make a strong appeal to the United States government for training support. As a result, the U.S. Department of State contracted with Daytop Village, Inc. to conduct extensive drug-free treatment training in Peru that included staff TC drug treatment institutional providers. This training was conducted in Lima, Peru. An extensive evaluation of this training was also conducted and was briefly described earlier[4].

The TC drug abuse treatment follow-up study presented below is an extension of the Johnson and colleagues[4] study described ealier[4]. Three research questions are posed for this follow-up study.

Question 1. What are the overall changes in illegal drug and alcohol use (to intoxication) of former clients of TC drug abuse treatment facilities in Peru?

Question 2. What are the predictors (treatment processes, capacity-building training exposure, and organizational/client characteristics) of illegal drug and alcohol to intoxication use among former TC drug treatment clients?

Question 3. Are the predictors (identified in question 2) moderated by contextual variables?

## Methods

A sample of 33 TC institutions and 509 former clients from a subset of the 76 TC facilities that participated in the earlier training impact study in Peru agreed to participate in the follow-up study We included former clients regardless of their discharge status if they stayed 30 days in treatment. While, on average, the attrition rate across the 33 participating TC institutions was 21 percent during the first 30 days, we believe the most accurate measure of treatment success should only include residents who stay 30 days or more. Thus, this 30-day eligibility selection criterion was used to only include clients who were really interested in dealing with their alcohol and/or drug problem. All data collection protocols were reviewed by a U.S.A. government approved Institutional Review Board in the Pacific Institute for Research and Evaluation, Inc.

### Research design

The study used a retrospective pretest (RPT) design with baseline collected retrospectively at a six-month follow-up assessment. Nimon and Allen[66] conducted an extensive review of RPT literature and found that since the seminal work of Howard, Ralph Gulanick, Maxwell, and Gerber[67], this evaluation design is reappearing more frequently in the literature. This design's strengths and weaknesses are discussed later. An attempt was made to collect baseline data on 30-day prevalence of alcohol and other drug use from clients' consent forms completed at intake. However, we discovered that there were large numbers of missing cases (about one-third of the data set). Comparable data on two of the five study outcomes (30 day use of any drug and alcohol to intoxication) were available from the consent forms at intake (n = 333) and RPT data at the six month assessment (n = 497) to determine differences in rates of use. Only a small difference for 30-day use of any illegal drug (87% vs. 90%) was found between the two samples. We did find higher percentages reported at intake vs. retrospective self-report at follow-up for nonuse of alcohol to intoxication (52% vs. 33%); however, in using retrospective reports, this is a more conservative estimate of change. Thus, we considered the use of a RPT design as valid for assessing change in use of global and specific drugs and alcohol to intoxication of former clients in the study TC facilities in Peru.

### Research setting and sample

This study was conducted in Peru in the capital city of Lima and five important cities of the Provinces (Iquitos, Tarapoto, Chiclayo, Trujillo, and Arequipa). The sample includes 33 Peruvian TC drug treatment facilities that identified themselves as therapeutic communities during the Drug-free Treatment (DFT) Training Evaluation study that was conducted in 1999[4]. This facility sample was 83 percent of eligible facilities for the study and data were collected from TC directors via a self-administrated questionnaire. From 33 TC drug treatment facilities, 879 clients remained in treatment for at least 30 days. Of this sampling frame, 509 clients were tracked six months after leaving treatment and interviewed (study retention rate = 58%). The remaining 370 former clients were difficult to track, because of four main reasons: (1) client address was not available or was incorrect (26%); (2) family reported client relapse and living in the street (21%); (3) family reported client at home and no relapse, but could not make contact (18%); and (4) family could not be not located or had rejected client (13%). Only 22 (6%) clients declined to be interviewed, which yields a cooperation rate of 94 percent.

We do not have comparative drug treatment studies in developing countries such as Peru to determine the adequacy of our response rates. However, as reported below, we assessed treatment success using a before treatment-six month follow-up analysis using three samples and found medium size treatment effects even when assuming that the entire baseline sampling frame including 370 former clients who could not be found at the six month follow-up were using drug and alcohol to intoxication 30 days prior to the six month follow-up period.

### Measures and data sources

Table 1 presents study measures, a description of the variables, their response or scale ranges, reliability scores, and data sources for the primary and secondary outcome measures, as well as moderating variables that were the main focus of the analysis.

**Table 1:** Study Measures and their Description

**Measures**^1^	**Description**^2^
**Outcome measures**	
Use of all illicit drugs 30 days prior to treatment (retrospective) vs. no use at the six month follow-up	1 = Yes, 0 = No
Use of PCB(Coca paste), cocaine, cannabis 30 days prior to treatment (retrospective) vs. no use at the six month follow-up	1 = Yes, 0 = No
Use of alcohol use to intoxication 30 days prior to treatment vs. no use at the six month follow-up	1 = Yes, 0 = No
**Treatment measures**	
Length of Stay (per 100 days)	.3 – 11
TC Model Implementation fidelity scale^3^	39% – 95%; (7 items; alpha = .65)
Treatment satisfaction^4^	1 = Very Satisfied, 2 = Somewhat Satisfied, 3 = Somewhat Dissatisfied, 4 = Very Dissatisfied (2 items; alpha = 0.69)
Training intensity	1 = 6–8 weeks of Daytop training in Peru v. 2 weeks of Daytop training in Peru, 0 = other
Director attended Daytop training in 1999	1 = Yes 0 = No
**Organization characteristics**	

Organization certified by Pervuvian Ministry of Health?	1 = Yes, 0 = No

Length of operation (years)	2 – 22

Number of paid full-time staff	0 – 32

Number of paid program professionals	0 – 17

Number of paid part-time and contract staff	0 – 11

Planned length of stay	12 months

	6 – 11 months

Number of clients served in 2002	8 – 580

Number of clients participating in Follow-up	2 – 33

Age of clients served	18 – 77 years

Classification of program	1 = TC, 0 = Non-TC

Number of Treatment Models Used	0 – 10

Percentage of clients that dropped out before 30 days	0 – 64

**Director characteristics**	

Ethnicity	0 = non-Mestizo, 1 = Mestizo

Gender	0 = Female, 1 = Male

Age	30 – 54

Education	1 = Primary School, 2 = Secondary School, 3 = Technical School Incomplete, 4 = Technical School Complete, 5 = Some University, 6 = University 4 year Degree, 7 = Higher than 4 year University Degree

Years in Organization	3 months – 22 years

Director attended Daytop training	1 = Yes, 0 = No

Director attended other TC training	1 = Yes – one or more, 0 = None

Recover Alcoholic or Drug Addict?	1 = Yes, 0 = No

Consult with staff before new policies are implemented	0 = Never, 1 = Rarely, 2 = Sometimes, 3 = Often, 4 = Very Often

Directly involved in clients' treatment plan	1 = Rarely, 2 = Sometimes, 3 = Often, 4 = Very Often

Amount of time spent individually with each client	1 = One or more hours, 0 = Less than one hour

Use of Research	0 = Never, 1 = Rarely, 2 = Sometimes, 3 = Often, 4 = Very Often

**Former Client characteristics**	

Ethnicity	0 = non-Mestizo, 1 = Mestizo

Age	18 – 77

Education	1 = Primary School, 2 = Secondary School, 3 = Any Technical School 4 = Some University, 5 = University 4 year Degree or more

Employment Status	1 = Employed Part or Full Time, 0 = Not Employed

Marital Status	1 = Married, 0 = Not Married

Participation in other treatment	1 = One or Two Kinds, 0 = None

Perceived stigma5	0 = low, 6 = high (9 items; alpha = .80)

Client propensity score	Predicted covariate to adjust attrition effect

Note: ^1 ^Imputed missing values were used that were based on an EM algorithm in the SPSS: Missing Values Analysis program[71]. ^2^Unless noted as a multiple-item scale, the measure is a single item. Factor analyses found all scales to be unidimensional. ^3^Sample item: "Some of the practices on the following list are part of a Therapeutic Community morning meeting and some are not. I'll begin with the first statement on this list. Please tell me whether or not this statement is true or false about how morning meetings were practiced in (INSERT TC NAME). '*Our morning meeting had two parts: one part for taking care of "business" issues and another part for taking care of "clinical" issues.'*" ^4 ^Sample Item: "How satisfied are you with the progress you made while you were in the program? Would you say... ^5 ^Sample item: "Most people who know I am a former alcohol or drug addict willingly accept me as a close friend."

Treatment success was defined as the presence or absence of 30-day use of illegal drug and alcohol to intoxication use 30 days before treatment with no 30-day illegal drug or alcohol use to intoxication at the six months assessment after treatment, respectively. We created dichotomous change scores (1 = use at baseline [retrospective measure] and no use at follow-up; 0 = other) for change in use of illegal drugs or alcohol to intoxication. There were 48 clients who did not report any drug use for retroactive baseline and follow-up. In addition, 148 clients did not report any alcohol intoxication prevalence for both baseline and follow-up. However, those clients are included in the analysis with outcome measures coded as 0 to be in the reference group, which is a more conservative approach.

The six key independent variables of interests were selective treatment processes and capacity-building training exposure that were expected to predict change in the drug and alcohol use outcomes. These variables are a combination of single items and unidimensional scales with the alpha reliabilities listed in Table 1 along with a sample item for each scale. These variables include length of stay, implementation fidelity, treatment satisfaction, Peru-based TC training intensity, director's participation in other training, and whether the TC director attended Daytop training. Length of stay was measured by a continuous variable where each unit equals 100 days in treatment – 1 to 10. There were 12 cases that exceeded 1,000 (considered outliers) so they were Windsorized to a value of 10. Implementation fidelity is a scale in which five to seven questions are asked about five important TC tools to ascertain the correctness of implementation. These tools were morning meetings, encounter groups, static groups, learning experiences (sanctions targeting a behavior or attitude change), and vocational skills. Daytop International assisted in constructing this scale. Melnick and De Leon's[68] research developed a survey of essential elements questionnaire (SEEQ) that taps congruence with the ideal TC model. However, the validated TC essential elements instrument was too long for the Peru study. A modified version that was validated for a similar TC treatment evaluation in Thailand found that the implementation fidelity focusing on TC tools was a stronger predictor of treatment success than the modified version of the SEEQ[69]. Treatment satisfaction was a two item scale about the client's self-reported satisfaction with the progress made and the outcome of the treatment experience. TC training intensity was a single dichotomous variable consisting of TC facilities that received six to eight weeks of in-country Daytop training vs. TCs that only received two weeks of in-country Daytop training. Director's TC training other than the in-country Daytop TC training was measured as a dichotomous variable one or more other TC training vs. none. TC-director-attended Daytop training was also a dichotomous variable coded as attended some or all of the training vs. none of the training.

The remaining variables at both the client and organizational level are covariates or moderators included in the multivariate analysis models. A missing value analysis was conducted for multiple items included in scales for client level variables and EM estimates for missing values were imputed[70,71]. We conducted a sensitivity analysis to determine how much the imputation of missing data in particular might affect the final results. These results with and with out the imputations were similar. More details of these measures, including scale alpha reliabilities and number of items, are reported in Table 1.

### Data collection

Data collection was conducted by a Peruvian partner (SERPA) in collaboration with U.S. investigators of Pacific Institute for Research and Evaluation, Inc. (PIRE) between January 15, 2003 and October 31, 2004. In-person interviews of the 33 directors occurred in January and February 2003 and of the 509 former TC clients between October 2003 and October 2004. Directors were asked to refer to records to answer client related questions, if they were not sure. Eligible follow-up study participants were identified as clients/residents who were already in treatment a minimum of 30 days as of January 15, 2003, or who were admitted after that date and who stayed a minimum of 30 days in the program. Study participants were informed that they were participating in the evaluation and they signed a written informed consent informing them of the goals and procedures of the study. One copy was also given to the client.

All subjects were recruited and interviewed by native Spanish-speaking interviewers who received special interview training, which PIRE staff observed. In-person interviews with item responses on cue cards were conducted at the former client's current place of residence in Lima and surrounding provinces of Peru. We used a drug testing procedure involving a random sample of 8 percent of the sample to increase the accuracy of the self-reported drug use data. We advised subjects that they might be randomly selected for a urine specimen that would be tested for the presence of marijuana or cocaine; but, their participation was completely voluntary. Respondents were not notified of the drug testing results, because the data from the tests were only used as a validity check on the self report data provided by those randomly selected for testing. As such, we treated all respondents equally in not providing drug testing results to respondents.

After completing the interview, a sealed envelope was opened that contained a label indicating whether the subject's ID number had been randomly selected for the urine specimen test. An analysis of the test results found high congruence between the drug test results and the self-reported drug use data collected during the follow-up interview, (i.e., 96% congruence). In addition, the analysis found no significant differences in self-reported drug use between respondents who were tested and those who were not, indicating that strategy was successful.

### Statistical analysis

The final analyses included a combination of data analysis methods, which included tests of significant differences between dependent proportions and Hierarchical Generalized Linear Modeling (HGLM) with appropriate statistical tests. The comparisons between dependent proportions compared the proportion of former clients who indicated using illegal drugs or alcohol to intoxication 30 days prior to treatment in comparison to 30 days prior to the six month follow-up interview. A dependent groups t-test using the binomial approximation to the variance determined statistical significance[73]. We calculated effect sizes by converting proportions with accompanying standard deviations to a Cohen's d, where a small effect size equals .20, a medium effect size equals .50, and a large effect size equals .80[72]. These effects sizes represent differences in standard deviation units using the normal distribution. We also conducted the same analysis on an expanded sample of 76 former clients whose parents reported that they had relapsed (65%) and the entire sample of eligible former clients (879). While assessing change in dichotomous outcomes traditionally uses a nonparametric statistical test, such as the McNemar test, we decided to use the parametric alternative, as we have an adequate sample size and the parametric alternative is justified under the central limit theorem[72]. Further, this inferential test lends itself to the calculation of a Cohen d effect size. Cohen's d has established qualitative effect size intervals of small, medium, and large[74] that we have found policy makers can easily interpret. While this test is traditionally evaluated using the normal standard deviate, we chose the slightly more conservative option of evaluating the test statistic using the t distribution.

In preparation for the HGLM analysis, first, we constructed valid and reliable scales, indexes, and single item measures. Second, we identified statistically significant covariates from a larger set of 26 potential covariates that were entered in a step-wise regression, predicting the outcome variables one at a time, using criteria of p < .20 to enter and p > .25 to be removed

The HGLM analytical procedure is appropriate in this study because data are nested in nature: the individuals (former residents) are nested in the 33 organizations (TC treatment facilities). HGLM adjusts for variation of dependent variables at the organization level, thus providing a more precise estimate of statistical coefficients at the client level. This model technique is explained in detail in Bryk, Raudenbush, and Congdon[75] and Raudenbush and Bryk[76], including all assumptions, techniques of estimation, and other statistical information. The basic concept behind hierarchical modeling is similar to that of logistic regression. At the client level (also referred to as level 1 in this study), the analysis is similar to that of logistic regression: the outcome variables in Table 1 are predicted by one or more level 1 variables plus an intercept with the treatment variables as the key independent variable of interest. At the TC level (level 2), the level 1 slope(s) and intercepts become dependent variables being predicted by level 2 TC characteristics variables.

Both predictors' main and moderating effects (also referred to as interactions) on the outcomes were assessed through a series of regression analyses to obtain a final equation with stable coefficients for each outcome. The moderating effects were determined by multiplying two variables producing a product term. If one or both of the variables were a continuous metric, they were centered prior to the multiplication. Dichotomous indictors were effect coded. Plots of the interactions against each outcome were assessed to confirm the direction of each statistically significant moderating effect.

Statistical significance was determined using a two-tailed test of significance with the level of significance set at alpha = .05. Effect size for the HGLM results were calculated for the point-biserial correlation with a small effect size equaling .10, medium effect size equaling .24, and large effect size equaling .37[74].

## Results

### Description of TCs, directors, and clients

#### Organizational (facility) Characteristics

A majority (64%) of the facilities had been certified by the Peruvian Ministry of Health; and the average length of time in operation was 10.2 years. The average number of paid full-time staff was 4; the average number of paid program professionals was 2.3 and part-time or contract staff was 2. The number of clients served in 2002, as reported by the DAT directors, ranged from 2 to 580 with a mean of 127 and the number of former clients participating in the follow-up study ranged from 2 to 33 with a mean of 15.4. The vast majority (94%) of the DAT facilities served adult clients and two-thirds (66%) also served clients 17 years of age and younger. The majority of the DAT facilities (58%) reported that their planned length of stay in treatment was 12 months and nearly one-fourth (24.2%) reported a planned length of stay of 6 to 11 months. The proportion of clients reported by the directors as dropping out before completing 30 days of treatment ranged from 0 to 64 percent across the facilities (mean = 21%). A report of no drop-outs during the first 30 days is unusual in the U.S.; however, four Peruvian TC facility directors did report zero dropouts. Thus, on average, 79 percent of the client population was eligible to participate in the study. In addition to the TC treatment model, on average, the DAT facilities employ 6.5 other treatment models (e.g., AA, Tough Love, behavior modification).

#### TC Director Characteristics

A majority of the TC directors reported they had attended the Daytop training (70%) and approximately three-fourths (76%) had attended other training on substance abuse treatment. A large majority (78.4%) of the TC directors classified themselves as Mestizo with the remaining 21.6% of the directors representing white (12%), black (6%), Indian (1%), Asian (.6%), and multi-racial (2%) groups. All of the study institutional directors were male (100%), the mean age of the sample was 44 years old, and on average, technical school was the average level of education they had completed. A majority of the TC directors (76%) were recovering alcoholics or drug addicts and had been with the TC facility, on average, for 8.5 years. On a scale of 0 (never) to 4 (very often), on average (2.51), DAT directors reported that they "sometimes" or "often" used research in their institutional decision making. On average, the TC directors reported that they consulted with their staff "often" before new policies were implemented. TC directors reported they were involved in clients' treatment planning "often" to "very often"; on average, 70 percent spent one or more hours per week individually with each client.

#### Former Client Characteristics

Nearly four out of five (79%) of the former TC clients described themselves as Mestizo and the final sample was 100% male. The ages of the former clients ranged from 18 to 76 with a mean age of 34. On average, secondary school (high school) was the highest level of education completed. Most (77%) were not married. One out of five (20%) had participated in at least one other treatment program since leaving the institution. Excluding the 12 former clients who reported having been in the DAT program for longer than three years, the actual average length of stay in treatment was slightly more than six months. On average, former clients reported being "somewhat satisfied" with both the treatment and services they received, as well as with the progress they made.

### Change in 30-day substance use six months after treatment

Question 1: What are the overall changes in drug and alcohol use to intoxication of former clients of TC drug abuse treatment facilities in Peru?

Former clients' 30-day use of illegal drugs and alcohol to intoxication before treatment was collected retrospectively at the time of the six-month follow-up interview of 497 former clients with complete data from 33 treatment facilities and compared to their self-reported use at a six-month assessment. We also report the analyses using an expanded sample that added 76 former clients whose family reported that they had relapsed (Table 2). These analyses of 30-day use change by substance shows statistically significant reduction for any illegal drug, specific drugs, and alcohol to intoxication. The percentage change for any 30-day illegal drug use for the client reported sample was 56 percent and was 49 percent when we added the 76 former clients (65% of the study population) whose family reported a relapse. These are large treatment effects (d = 1.12 and .98 respectively). When examining specific drug use, statistically significant reductions in 30-day use of PCB (coca paste), cocaine, cannabis, alcohol, and alcohol to intoxication were also found. The magnitude of the treatment effect ranged from medium (d = .54) to large (d = .82) for the self reported sample and medium effects (d = .48 to .72) for the self or family reported sample, which are medium treatment effects. Finally, the prevalence of 30-day alcohol use to intoxication reduced significantly as well, regardless of the sample (d = .67; .62).

**Table 2:** Overall change in 30-day substance use (prevalence) before and six months after treatment (N = 497)

Type of Substance use	Before Treatment		6 months after Treatment		Difference^3^		Effect Size^4^	
	Self reported^1^	Self or Family reported^2^	Self reported^1^	Self or Family reported^2^	Self reported^1^	Self or Family reported^2^	Self reported^1^	Self or Family reported^2^
Illegal Drug use	90%	91%	34%	43%	56%***	49%***	1.12 (Large)	.98 (Large)
PCB (Coca Paste) use	62%	67%	22%	33%	40%***	35%***	.82 (Large)	.72 (Medium)
Cocaine use	30%	39%	8%	20%	22%***	19%***	.52 (Medium)	.48 (Medium)
Cannabis use	37%	45%	13%	24%	24%***	21%***	.53 (Medium)	.49 (Medium)
Alcohol use to intoxication	68%	72%	33%	42%	35%***	31%***	.67 (Medium)	.62 (Medium)

Note: *p ≤ .05, **p ≤ .01, ***p ≤ .001; ^1 ^Includes Self reported relapse (n = 497); ^2 ^Self or Family indicated relapse (n = 573); ^3^Standard deviations may be calculated from percentages using the binomial approximation to the variance, where SD = (p(1 – p))^2^; ^4^Effect Size: Proportions converted to Cohen d statistic with a small effect size equals .20, a medium effect size equals .50, and a large effect size equals .80[73]

We also conducted an analysis that assumed the 370 former clients not interviewed at follow-up would be using one or more illegal drugs and alcohol to intoxication (n = 879). This conservative analysis strategy showed treatment effects not reported in a table to be medium for illegal use and alcohol to intoxication reductions (d = .68, .46 respectively).

In summary, these results show that former clients of the drug abuse treatment under study improved their quality of life after treatment. While definitive conclusions that the treatment produced positive results cannot be made without a control group, the changes are sufficiently large to conclude that treatment more than likely produced the results. Further conducting the analysis using two additional samples (65% and 100% of the study population) further validated the conclusion that there was treatment success using all five outcomes.

### Predictors of successful TC treatment in Peru

Question 2: What are the predictors (treatment processes, capacity-building training exposure, and organizational and client characteristics) of drug use among former TC drug treatment clients?

In this analysis we examined a number of predictors of change in the outcome variables – treatment success as measured by favorable change in 30-day illegal drug use and alcohol use to intoxication before treatment and six months after treatment, including clients who graduated and clients who left treatment early. The predictors included a set of treatment and capacity-building training variables as well as TC facility, director, and client characteristics. (See Table 1 for possible predictors and Table 3 for the specific predictors in the final HLM regression equations).

**Table 3:** HGLM regression of illegal drug and alcohol use to intoxication onto treatment processes, capacity-building training, client and organizational predictors (n = 497)

	**Decrease in Illegal Drug Use**			**Decrease in Alcohol to Intoxication**		
**Predictors**	**Odds Ratio**	**Effect Size (r)**	**T ratio**	**Odds Ratio**	**Effect Size (r)**	**T ratio**

**Treatment**						

TC length of stay	1.19	0.09	2.04*	0.95	-0.02	-0.36

TC Model Implementation fidelity	1.08	0.14	3.03**	0.97	-0.03	-0.74

TC treatment satisfaction	1.01	0.00	0.07	0.62	-0.12	-2.66**

**Capacity-building Training**						

TC training intensity	1.15	0.03	0.7	1.2	0.06	1.2

TC staff participated in drug treatment training	1.44	0.08	1.67	1.14	0.03	0.68

TC director Daytop training in 1999	1.46	0.04	0.89	0.86	-0.03	-0.62

**Client characteristics**						

Client age	1.14	0.12	2.62**	1.13	0.11	2.43*

Client education	0.83	-0.07	-1.59	0.91	-0.04	-0.8

Client employment status	0.58	-0.04	-0.8	0.94	-0.02	-0.46

Perceived stigma	0.54	-0.19	-4.27**	0.12	-0.12	-2.67**

Client ethnicity	0.89	-0.04	-0.92	1.06	0.02	0.49

Client attended other treatment	1.14	0.03	0.59	1.56	0.09	1.91

**Organizational characteristics**						

Percentage of clients that dropped out before 30 days	1.01	0.06	1.22	1	-0.02	-0.51

Institution certified or not	0.87	-0.04	-0.82	1.17	0.04	0.77

Director collaborated with staff	1.42	0.08	1.64	0.9	-0.03	-0.58

Director's time spent with clients	0.41	-0.13	-2.75*	0.82	-0.04	-0.84

Director's ethnicity	0.96	-0.01	-0.26	0.85	-0.06	-1.35

Director's age	1	0.01	0.12	0.96	-0.07	-1.45

Director a recovering addict	1.15	0.03	0.65	1.16	0.02	0.45

Intercept	.00	-.08	-1.63	8.21	0.03	.553

**Interactions**						

Stigma × implementation fidelity	---	---	---	1.03	0.11	2.39*

Client age × Implementation fidelity	.99	-.12	-2.53*	.99	-.08	-1.75

Note: D.F. = 473; An attrition bias correction score [not in table] was used as a control variable, which increases the generalizability of these results; *p ≤ 0.05, **p ≤ 0.01. Effect size = point – biserial correlation with a small effect size equals .10, a medium effect size equals .24, and a large effect size equals .37[73]. Length of stay coefficient is scaled to show increase in logistic coefficient for every 100 day increase in length of stay. TC = Treatment Center.

Some results were consistent with expectations on at least one of the two outcome measures of treatment success. Table 3 shows that the longer the stay in treatment, the more treatment success for illegal drug use (i.e., a larger proportion of former clients not reporting 30-day illegal drug use in comparison with their reported 30-day use at baseline) (See Table 3). There was no significant relationship between length of stay and treatment success for the use of alcohol to intoxication. We also found that as the fidelity of use of TC tools increased, the greater the treatment success (See Table 3). In addition, higher level of stigma (i.e., perceived negative reactions from members of the community – for example, close friends, employees, girlfriends) was a consistent predictor of less treatment success for both illegal drug use and alcohol use to intoxication (See Table 3). This result suggests that reducing negative community reaction (i.e., stigma) may lead to a higher proportion of clients who report a decrease of drug use and alcohol use to intoxication. Client age is shown to have positive impact on decrease in both use of illegal drugs and alcohol use to intoxication (See Table 3). That is, the older the former client, the more successful the treatment.

Regarding inconsistent results, Table 3 also shows that the greater clients' treatment satisfaction, the less treatment success (i.e., a smaller the proportion of former clients who reported not using alcohol use to intoxication (See Table 3); whereas treatment satisfaction among clients with illegal drug problems had no effect. We also found that among TC facilities where the directors reported spending more time with clients, clients were less successful in treatment. None of the training capacity-building training variables were singly related to treatment success.

Question 3: Are the predictors (identified in question 2) moderated by contextual variables?

Table 3 also presents the moderating (or interaction) effects of treatment processes and capacity-building training exposure, and contextual factors on illegal drug use and alcohol use to intoxication. The table shows among those TCs with higher implementation fidelity, former clients with lower stigma have more success in dealing with alcohol use to intoxication (See Table 3). This positive moderating result is in addition to positive effects of the higher implementation fidelity and lower stigma main effects. For the illegal drug use, more treatment success occurred among younger former clients who were in TC facilities with higher implementation fidelity (See Table 3). This is a positive outcome in that, while older clients tend to achieve better treatment success (see main effect of client age variable), the combination of younger age and higher fidelity also contributes to better treatment success. There were no other statistically significant moderating effects on treatment success that were interpretable.

## Discussion

This study found that former clients in Peru who received drug and alcohol treatment in facilities using the TC model reported substantial positive change in use of illegal drugs and alcohol to intoxication at a six-month follow-up. The extent of change is greater than has been reported in other drug treatment program evaluations, including the Treatment Outcome Prospective Study (TOPS) [7,12], the Drug Abuse Treatment Outcome Study (DATOS)[77], and the National Treatment Improvement Evaluation Study (NTIES)[78]. Likewise, the amount of positive change in outcomes in Peru is also larger than National Treatment Outcome Research Study (NTORS)[79,33]. Because these other studies are not comparable on data collection timeframes or the substance use recall period, we can not conclude greater drug and alcohol treatment success in Peru than elsewhere. However, these results are similar to a more recent evaluation of TC treatment success (30-day use of illegal drugs) in Thailand that used a pre-post design with baseline data collected prior to treatment – reduction = -63 percent[69].

Regarding predictors of drug treatment success, we found that high implementation fidelity produced more treatment success. These results support the meta-analysis of Prendergast, Podus, and Chang[44], who found that well-implemented TC drug abuse treatment and outpatient drug-free programs correlated with more positive behavioral outcomes. In the Peru research we also found that the importance of implementation fidelity in predicting treatment success was enhanced among younger clients. That is, while older clients tend to achieve better treatment success (see main effect of client age variable), the combination of younger age and higher fidelity also contributes to better treatment success. Hansen and colleagues[49] also found higher program fidelity moderates drug prevention success.

Clients' higher level of stigma after leaving treatment was a consistent predictor of less treatment success in the Peru study. These results indirectly support the earlier work of Falck and colleagues[55], who found stigma to be associated with cocaine use, and Semple and colleagues[56], who found stigma to be a significant predictor of depressive behavior. An additional value of this research in Peru is that TC implementation fidelity moderates the effects of higher stigma on treatment success. That is, among TC facilities that implemented the TC tools more correctly, former clients report more treatment success regardless of higher stigma in the community after leaving treatment.

Consistent with prior research [26,11,28], length of stay in the treatment program correlated with treatment success for impacting illegal drug use, although this relationship was weak. When defining treatment success as reduced use of alcohol to intoxication, we found no relationship with length of stay. That is, if a client stayed 30 days, which was part of the eligibility requirement of this study, length of stay beyond 30 days made no difference in the proportion of former clients reporting 30 day alcohol use to intoxication in comparison with retrospective baseline 30 day use. It may be when heavy alcohol use is a problem, client stay in treatment as long as is needed.

Further, unlike prior studies that found a positive correlation between treatment satisfaction and success after treatment, the Peru study found that less treatment satisfaction led to higher treatment success. This result in Peru may be plausible in that lower satisfaction may result from more rigorous implementation of the TC model, which contributes to more treatment success. It is interesting to note that TC staff informed the Daytop team during the course of training that they were experiencing resistance from clients as they made changes to the implementation of TC tools and methods, making the program more challenging for the clients.

The treatment study in Peru is not without methodological controversy. First, the response rate of 58 percent is less than is reported in the U.S. However, since there has been little treatment evaluation conducted in developing countries, we believe the U.S. standards should not be applied. Data collection in developing countries is much more difficult with limited treatment facility infrastructure to support baseline data collection and a satisfactory tracking system. While generalization from a treatment sample to a treatment population is important to determining success, we did conduct comparative analyses that included former clients not interviewed in the sample and assumed they were users. Thus, they were counted as treatment failures in the analyses. These results of drug and alcohol use reductions were lower than those that only included self reports, but still they showed treatment success.

Second, we used a retrospective pretest treatment (RPT) only design in place of a traditional pre-post treatment only design. Lamb[80] declared the RPT design as imperfect but useful. These authors and others have discussed the strengths and weaknesses of this design. For this study, the major strength is the design allows collection of data when pre-testing is impossible. The major weakness is problems associated with memory and recall. We believe the comparison of our retrospective pretest data with the consent data at intake demonstrates the validity of our collection of baseline data retrospectively. That is, our comparative analysis showed that retrospective reported baseline data were, in most cases, lower prevalence than consent reported use at intake.

## Conclusion

The unique contribution of this study is that the results suggest attention should be placed on the importance of implementing the TC drug abuse treatment model with high fidelity, especially in connection with lower client stigma as perceived by the former resident. The results also strongly suggest that TC drug abuse treatment programs should incorporate follow-up activities that attempt to neutralize community negative reactions (perceived stigma) independent of other factors. For example, in Peru and Brazil, there are drug abuse treatment programs among street children that incorporate a follow-up strategy that may positively impact stigma[81]. High implementation of TC tools and principles, along with implementation of stigma reducing follow-up strategies is not a panacea for treatment success of its clients. However, this research suggests that these organizational actions may help improve the quality of life of TC treatment former clients.

## Competing interests

The authors declare that they have no competing interests.

## Authors' contributions

K. Johnson directed the preparation of the manuscript and prepared text for parts of the methods and results and all of the discussion and conclusions. Zhenfeng Pan conducted the initial HGLM analysis and prepared the results text, Linda Young prepared the introduction, Jude Vanderhoff prepared the data, conducted the descriptive analysis and prepared the reference section, Steve Shamblen conducted additional analyses and prepared the results inserts. Thom Browne provided inserts in the introduction and reviewed of the entire manuscript. Ken Linfield conducted the preliminary SPSS analysis of the study and Geetha Suresh also conducted aspects of the revised analysis plan.
